# Screening Questionnaire for Vitamin D Insufficiency in Children with Obesity

**DOI:** 10.3390/children9111685

**Published:** 2022-11-02

**Authors:** Valeria Calcaterra, Hellas Cena, Ginevra Biino, Roberta Grazi, Giulio Bortoni, Valentina Braschi, Chiara Elena Tomasinelli, Laura Schneider, Gianvincenzo Zuccotti

**Affiliations:** 1 Department of Internal Medicine and Therapeutics, University of Pavia, 27100 Pavia, Italy; 2 Pediatric Department, “Vittore Buzzi” Children’s Hospital, 20154 Milano, Italy; 3 Laboratory of Dietetics and Clinical Nutrition, Department of Public Health, Experimental and Forensic Medicine, University of Pavia, 27100 Pavia, Italy; 4 Clinical Nutrition and Dietetics Service, Unit of Internal Medicine and Endocrinology, ICS Maugeri IRCCS, 27100 Pavia, Italy; 5 Institute of Molecular Genetics, National Research Council of Italy, 27100 Pavia, Italy; 6 Department of Biomedical and Clinical Science, University of Milano, 20157 Milano, Italy

**Keywords:** VitD, pediatric obesity, screening tool, insufficiency, questionnaire

## Abstract

Non-invasive screening tools to identify children at high risk of vitamin D (VitD) deficiency are proactive measures in preventive care. Recently, a validated questionnaire (Evaluation dEficieNCy Questionnaire, EVIDENCe-Q) for identifying newly diagnosed VitD-insufficient adults has been developed. We tested the EVIDENCe-Q modified for children with obesity and evaluated the correlation between VitD and questionnaire scores to adapt this tool to the pediatric population. We enrolled 120 children with obesity (BMI ≥ 2). Clinical evaluation and VitD levels were considered. The modified EVIDENCe-Q included information regarding factors affecting control of VitD, with scores ranging between 0 (best) and 36 (worst). VitD and adiposity indices were inversely correlated. The threshold values for identifying severe deficiency (<10 mg/dL), deficiency (<20 mg/dL) and insufficiency (<30 mg/dL) were scores of 21, 19 and 23, respectively. According to those thresholds, the prevalence of severe deficiency, deficiency and insufficiency was 47.5%, 69.2% and 23.3%, respectively; the best accuracy was obtained with a questionnaire score cut-off of 19 for the VitD deficiency level. A novel simple screening tool such as the modified EVIDENCe-Q would be useful in clinical practice to identify potential cases of hypovitaminosis D and select at-risk patients. Considering the limited accuracy and specificity of our results, for the pediatric population a dedicated tool should be created. Phases of childhood and the role of adipose tissue could be considered in the definition of a questionnaire intended for pediatric patients with obesity.

## 1. Introduction

Vitamin D (VitD) is recognized to be a hormone with pleiotropic action on many organs and tissues, and its deficiency may be associated with many disease outcomes, including bone and extra-bone outcomes, such as osteopenia, osteoporosis, cardiovascular diseases, cancer, autoimmune diseases, infectious diseases, chronic obstructive pulmonary disease and cognitive deficits [1,2]. The two major forms of VitD are VitD2 and VitD3. VitD2 (ergocalciferol) is largely human-made and added to foods, whereas VitD3 (cholecalciferol) is synthesized in the human skin from 7-dehydrocholesterol and is also consumed in the diet via the intake of animal-based foods [3]. Both forms are converted to 25-hydroxyvitamin [25(OH)D], which may function as a biomarker of exposure. However, the extent to which 25(OH)D values serve as a biomarker of effect is not fully established [4,5,6,7,8].

Hypovitaminosis D and deficiency have a high prevalence worldwide. The overall prevalence of insufficiency in Europe has been estimated at around 13% [9]. In the UK, according to National Diet and Nutrition Survey data, 10% of children from 4 to 10 years and 26% of children from 11 to 18 years present serum 25(OH)D levels below the cut-offs [10]. Girls seem to be at higher risk than boys, with 13% of girls between 4 and 10 years as well as 39% of girls between 11 and 18 years presenting serum deficiency [11]. In Italy, the prevalence of hypovitaminosis D in pediatrics is above 50%, in particular during adolescence [11,12].

Furthermore, in children and adolescents, body mass index, ethnicity, sun exposure and seasonality impact are the key factors affecting VitD status [11,12,13].

In particular, pediatric subjects with overweight and obesity are at risk of VitD insufficiency. As reported by Yao [14] in a meta-analysis including 15 manuscripts, a significant difference in insufficiency prevalence among patients with obesity was observed, with an OR of 3.70 (2.33–5.06). Sequestration of VitD into fat likely plays a significant role in reducing the amount that can be presented to the liver for 25-hydroxylation, with a consequent reduction in its serum concentration [14].

Several cut-offs have been reported in order to define deficiency by means of serum 25-hydroxy (OH) as a biomarker, but in pediatrics, the threshold definition is complex [8].

According to the Italian Pediatric Society and the Italian Society of Preventive and Social Pediatrics, a serum 25-hydroxy (OH) level of below 10 ng/mL is defined as severe deficiency, below 20 ng/mL as deficiency and 20–29 ng/mL as insufficiency, while levels higher than 30 ng/mL are defined as sufficiency [8].

Currently, the prevention of VitD insufficiency requires costly and uncomfortable blood testing, and VitD supplementation is often prescribed empirically with the risk of leading to a liposoluble vitamin overload.

Indeed, in children and adolescents, 600–1000 IU/day VitD supplementation is recommended in the presence of the risk of deficiency. Moreover, considering drug–nutrient interactions, children on oral corticosteroids, anticonvulsants, antimycotics and anti-retroviral medications are recommended at least 2–3 times higher vitamin D doses than other children, equivalent to 2000–4000 UI/ day for at least 6–8 weeks [8,15]. In case of permanent risk factors, continuous supplementation is recommended. In addition, in case of reduced sun exposure, supplementation should last throughout the winter period, but when the risk factors are persistent, supplementation should last all year [15,16].

In case of asymptomatic deficiency, in order to obtain sufficiency, the supplementation suggested is around 2000 IU/day or 50,000 IU/week for 6–8 weeks, followed by a re-evaluation of the serum concentration at the end of the treatment [16]. However, despite the need to reach an adequate level of plasma vitamin D in children with overweight and obesity, no consensus on the supplementation dose and length of treatment has been reached [17].

Currently, the prevention of VitD insufficiency requires costly and uncomfortable blood testing, while empiric VitD supplementation without testing might lead to a vitamin overload.

The authors hypothesized that children and adolescents with overweight and obesity would commonly have an inadequacy or deficiency of VitD and that a low-cost and non-invasive tool such as a questionnaire could help identify patients with the condition, with clinical application in daily practice.

In 2021, the EVIDENCe-Q project (Evaluation dEficieNCy Questionnaire) [18] headed by De Giuseppe et al. led to the development and validation of a questionnaire screening for Vit D deficiency comprising 20 items scoring from 0 (equivalent to the best status) to 36 (equivalent to the worst status), in order to evaluate the adequacy of vitamin D status in Italian adults.

This questionnaire showed a good inverse relationship with the gold standard level according to the “Italian Society for Osteoporosis, Mineral Metabolism and Bone Disease” [18,19], providing a useful, easy and unexpensive screening tool for clinicians in their daily practice, which is helpful in identifying subjects potentially at risk of VitD deficiency and avoiding unwarranted supplementation and/or costly blood testing.

Based on that study, the authors of this manuscript aimed to describe the use of a modified version of the questionnaire (modified Evaluation dEficieNCy Questionnaire) adapted to the pediatric population affected by obesity, considering that additional revisions may ameliorate its ability to predict VitD deficiency in children too.

## 2. Materials and Methods

### 2.1. Patients

We consecutively enrolled 120 Caucasian pediatric patients (59 females and 61 males, aged between 3 and 18 years) with obesity (BMI z-score ≥ 2) who were referred to the Vittore Buzzi Children’s Hospital by their general practitioner or primary care pediatrician. Children were referred between September 2020 and February 2022. Exclusion criteria were as follows: known secondary obesity conditions, use of any medications, concomitant chronic or acute illnesses, medications that interfere with VitD levels, micronutrient supplementation.

In all subjects, clinical evaluation and serum VitD levels were considered. 25(OH)D values (ng/mL) were routinely measured as part of the nutrition assessment in all children with obesity attending the endocrinological outpatient service.

The study was conducted in accordance with the Helsinki Declaration of 1975, as revised in 2008. The institutional ethics committee approved the protocol (Ethics Committee Milano Area 1; Register study 2020/ST/234 Date 29/07/2020; Prot. N 0030785 Date 12 July 2021). After being informed about the nature of the study, all participants or their responsible guardians gave their written consent. 

### 2.2. Methods

#### 2.2.1. Auxological Evaluation

In all participants, height (Ht), weight, pubertal stage and waist circumference were measured, and WC/Ht and BMI were calculated. Anthropometric measurements were performed as previously reported [20,21]. Pubertal stages were classified as follows: Tanner 1 = pre-pubertal stage 1; Tanner 2–3 = middle puberty stage 2; Tanner 4–5 = late puberty stage 3 [22,23].

BMI was calculated as body weight (kilograms) divided by height (meters squared). BMI was transformed into BMI z-scores using WHO reference values [24] and all subjects were classified as obese when the BMI z-score was ≥2.

#### 2.2.2. VitD Level Assessment

For all children, we considered 25(OH)D (ng/mL) serum levels that had been routinely determined using the commercial kits (Alinity i 25-OH VitD reagent kit, Abbott Park, IL, USA) on the ARCHITECT system, as reported in their medical records.

To evaluate the VitD status of each subject, we adopted the 25(OH)D cut-off values proposed by the Italian Pediatric Society and the Italian Society of Preventive and Social Pediatrics, classifying severe deficiency, deficiency, insufficiency and sufficiency as 25(OH)D levels of <10 ng/mL, <20 ng/mL, between 20 and 29 ng/mL and >30 ng/mL, respectively [8].

### 2.3. Questionnaire Design and Scoring

The previously developed self-administered 20-item EVIDENCE-Q (ClinicalTrials.govID: NCT04404842), which is used to screen VitD status adequacy in the Italian adult population [25], was considered and modified to be adapted to the pediatric population.

The self-administered 20-item EVIDENCe-Q [18] includes multiple-choice questions (13 dichotomous mode/7 polytomous mode) investigating factors affecting VitD level production/intake, absorption and metabolism. In particular, this questionnaire investigates the following: (i) geographical information on the place of residence (north, south or central Italy, as well as urban and peri-urban home area); (ii) skin phototype; (iii) regular outdoor physical activity (yes/no); (iv) exposure to sunlight for at least thirty minutes, specifying how many times a week (0–7 times) and if during the 10:00 a.m–15:00 p.m. slot (yes/no); (v) habitual use of sunscreen with a sun protection factor (SPF), specifying if the SPF ≥15 (yes/no/only during summer) and the frequency of use of sunscreen during sun exposure (one time; two times; three or more times); (vi) monthly use of UV tanning lamps (1 ≤ time/2–3 times/4–5 times); (vii) consumption of dietary sources of VitD; (viii) pathologies that interfere with the production and/or absorption of VitD (yes/no); (ix) medications that may interfere with nutrient bioavailability and/or requirements (yes/no); (x) use of multivitamin supplements or supplements containing VitD (yes; no).

The EVIDENCE-Q score ranges from 0 (best VitD status) to 36 (worst status). In particular, responses assuming behavior that does not lead to deficiency are assigned a score of zero, while those potentially leading to deficiency are assigned a score of 1 (if the response mode is dichotomous) or greater than 1, increasing by one unit for each answer that assumes a progressively worse behavior.

### 2.4. Statistical Analysis

Descriptive statistics with the mean and SD were used to depict the sample and the correlation between 25(OH)D serum values and BMI and clinical features, as well as between 25(OH)D serum levels and the questionnaire total score, which was calculated to analyze the relationship between two pairs of variables.

Analysis of variance (ANOVA) was utilized to analyze the score mean value in the 4 classes of VitD status (severe deficiency, deficiency, insufficiency, sufficiency).

Analysis of the ROC (receiver operating characteristic) curve was used to identify the threshold values of the questionnaire score, which is useful to discriminate between the subjects who are at risk of deficiency or insufficiency and those who are not.

STATA software version 16 (College Station, TX, USA) was used to perform all the analyses.

## 3. Results

Sample characteristics are reported in Table 1.

### 3.1. Demographic and Auxological Parameters and VitD

No significant differences were noted between the sexes in 25(OH)D levels (males 17.9 ± 6.76 vs. females 18.1 ± 7.87, *p* > 0.05). On the contrary, a significant decrease in VitD levels was noted between pre-pubertal and pubertal stages (Tanner 1: 19.6 ± 8.28; Tanner 2: 18.5 ± 6.39; Tanner 3: 14 ± 4.53; *p*-value ANOVA = 0.0041), without a difference between the sexes (*p* = 0.146).

As shown in Table 2, VitD serum values were inversely correlated with adiposity indices, including weight, BMI and WC; no significant correlation with W/H was revealed except for the WC/H ratio.

### 3.2. Questionnaire Score

No differences between genders or pubertal stages were noted according to the questionnaire score (*p* > 0.05).

The questionnaire scores according to serum status are reported in Table 3. The average score in the three subgroups was not different, considering serum levels of both 30 and 20 mg/dl as cut-offs for insufficiency (ANOVA *p*-value = 0.870 and 0.877, respectively; Table 3).

**Figure 1:**
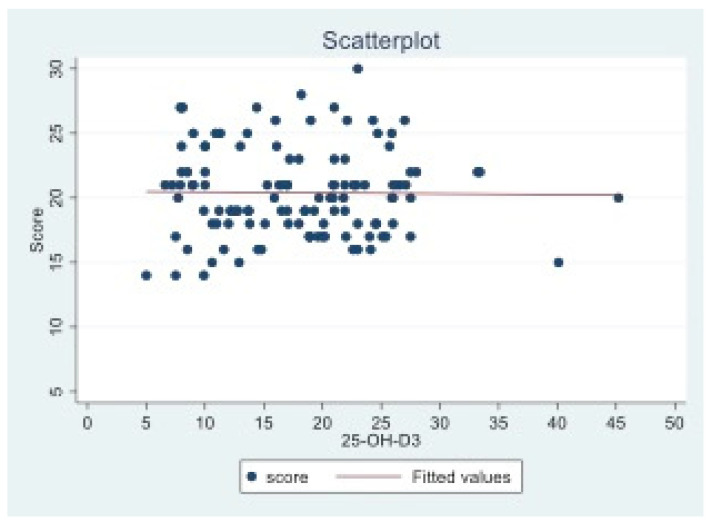
Scatter plot of questionnaire scores vs. VitD levels.

As shown in Figure 1, no linear correlation was observed between VitD levels and the questionnaire scores.

As reported in Table 4 and Table 5, a threshold value for the questionnaire score of 21 for severe deficiency (<10 mg/dL), a threshold value of 19 for deficiency (<20 mg/dL) and a threshold value of 23 for insufficiency (<30 mg/dL) were identified, using the ROC curve. The accuracy and specificity are limited. According to these thresholds, the prevalence of severe deficiency, deficiency and insufficiency was 47.5%, 69.2% and 23.3% of the study population, respectively; the best accuracy, with higher specificity, was obtained with a questionnaire score cut-off of 19 for VitD level deficiency (Table 5).

## 4. Discussion

VitD deficiency affects more than half the population of all ages. Hypovitaminosis D in pediatric age is a re-emerging public health problem all over the world, especially in Western countries. Lifestyle habits, current “epidemics” of obesity in children and adolescents and other preventable risk factors may play a crucial role in the VitD deficiency occurrence [25].

Considering that roughly 43 million children are estimated to be affected by obesity throughout the world, anyone could easily predict the size of the risk of vitamin D deficiency in this category of subjects, who are a vulnerable group for poor VitD status, showing a significant decrease in VitD levels in pubertal stages compared to pre-pubertal conditions, supporting the important role of puberty in VitD metabolism. Puberty, indeed, is a crucial time in the development of the bone mineral mass, with increased requirements in this period [26,27].

In our patients, VitD status, fat mass and adiposity indices, including weight, BMI and WC, were inversely correlated, as previously reported in adults [18,28]. The effect of excess body fat on VitD control is not fully elucidated, but evidence shows that increased adiposity leads to lower serum 25(OH)D values, and, conversely, weight loss reduces peripheral sequestration and enables higher 25(OH)D values [3]. VitD is absorbed with fats as part of chylomicrons and is taken up first by peripheral tissues that express lipoprotein lipase, such as adipose tissue and skeletal muscle. Moreover, increased metabolic clearance through augmented uptake, decreased bioavailability once deposited in adipocytes and/or VitD volumetric dilution due to a larger body mass have been hypothesized as possible effects [17,18,29,30].

Moreover, VitD deficiency appears to exacerbate the negative impact of obesity on the risk of many noncommunicable diseases such as type 2 diabetes, cardiovascular diseases, different types of cancer and respiratory diseases, but also communicable diseases [31,32]. Recently, there has been an exponential increase in studies of extra-bone actions of VitD, including immunomodulation and autoimmune disease development, as well as in chronic low-grade inflammation and endo-metabolic diseases, chronic respiratory conditions, dermatological diseases and cardiovascular pathologies [33].

VitD insufficiency is reported in children with overweight and pathological carbohydrate and lipid parameters [34]. Additionally, low VitD levels appear to be associated with insulin resistance, and hypovitaminosis D is considered one of the factors accelerating the development of insulin resistance, leading to prediabetes and diabetes [34].

The correction of VitD insufficiency is associated with attenuation of the related obesity dysmetabolism. Therefore, screening strategies and prevention interventions aimed at correcting VitD insufficiency may be useful in reducing metabolic and cardiovascular adverse events related to obesity.

A simple and inexpensive questionnaire to assess VitD insufficiency could offer a useful and easy screening tool for pediatricians in their daily practice to protect the health of children and adolescents.

According to De Giuseppe et al., the EVIDENCe-Q is an easy-to-use tool that demonstrates a good relationship with VitD serum levels, showing potential for strong support in clinical practice, helping to identify subjects potentially at VitD deficiency risk and to avoid unwarranted supplementation and/or costly blood testing [18].

In the present study, the authors tested a modified version of the EVIDENCe-Q, which was adapted to the pediatric population. The questionnaire, consisting of 20 multiple-choice questions, investigated the factors affecting the production, absorption and intake of VitD in a sample of 120 pediatric patients. Data on demographic information, habitual dietary patterns, state of health, pharmacological treatments, supplement intake, lifestyle habits, and the frequency and time of exposure to direct sunlight, depending on the season, and combined with the use of protective sunscreens, were collected and evaluated.

The threshold value for the questionnaire score to estimate VitD status in children and adolescents is different compared to adults [18]. Additionally, the accuracy of the instrument is lower than in adults. These data support the need to create a dedicated tool in different and specific population groups (e.g., infants, children, adolescents).

We failed to record a significant difference in the questionnaire score between males and females, as well as according to pubertal stages; moreover, the average score did not significantly differ consistently with the severity of VitD deficiency. In our pediatric population, the best cut-off to discriminate between deficient (<20 ng/mL) and sufficient VitD subjects was a score of 19 points, which identified more than 69% of the patients with VitD deficiency. Meanwhile, in the adult population, EVIDENCe-Q threshold values of 23, 21 and 20 were identified, respectively, for severe deficiency, deficiency and insufficiency, according to which the prevalence of severe deficiency, deficiency and insufficiency was 22%, 35.3% and 43.3% of the study population [18].

According to literature data, VitD treatment is recommended in all children with 25(OH)D serum levels <20 ng/mL; on the contrary, supplementation remains controversial for levels between 20 and 30 ng/mL [8]. Thus, even though the results of the ROC analysis were not fully satisfactory, these threshold values can offer important and immediate support for clinicians in their daily practice, in order to identify subjects potentially at VitD deficiency risk and to avoid unwarranted supplementation and/or costly blood testing.

The modified EVIDENCe-Q offers additional strengths, since it is quick and easy to use, and it also investigates supplementation considering the treatment dose and duration in relation to lifestyle and dietary habits and the clinical condition. Thus, this questionnaire may also represent a good tool to monitor VitD status, especially since reassessment is recommended no earlier than six months post-VitD supplementation, with the exception of special cases [35,36]. Finally, by investigating lifestyle and nutrition habits, this tool may offer educational support to encourage children and their families to adopt healthier dietary habits and lifestyles.

The strengths of our study include the testing of individuals of both genders (59 females and 61 males), from ages 3 to 18 years, with obesity (BMI z-score ≥ 2), and the uniform measurement of serum 25(OH)D using a single assay; however, study weaknesses also exist. Firstly, the authors are aware that the relatively small sample size does not reach statistical power necessary to significantly extrapolate the results for the whole population and may limit the results of ROC analysis. However, the results are undoubtedly worthy of further studies extending the data collection and increasing the quality of research. Secondly, considering the differences noted on comparing the results obtained from the adult version of the EVIDENCe-Q, the authors are planning to enhance the instrument structure to better meet pediatric needs and test the instrument in different phases of childhood to optimize the accuracy.

Additional studies are needed to refine the current questionnaire in order to create a dedicated tool for a pediatric population who may benefit from serum 25(OH)D measurement. Ideally, a larger study performed in different phases of childhood, such as infants, children and adolescents, with a questionnaire modified from the one presented herein is recommended to provide a “score” with higher sensitivity and specificity for vitamin D deficiency, in order for this questionnaire to become a useful tool in clinical practice. Considering the role of adipose tissue, additional item for the score could be necessary in pediatric patients with obesity, also considering metabolic complications.

## 5. Conclusions

Considering the high prevalence of VitD deficiency in children and adolescents with overweight and obesity, a novel simple screening tool such as the modified version of the Evaluation dEficieNCy Questionnaire would be useful in identifying potential cases of hypovitaminosis D and selecting patients at risk who require blood testing and personalized supplementation, which will lead to sure benefits if researchers confirm the importance of VitD in bone and extra-bone health. Phases of childhood may represent a crucial point to consider for a pediatric dedicated tool. Additionally, specific items for patients with obesity could be included.

## Figures and Tables

**Table 1:** Clinical and anthropometric characteristics of the subjects according to gender (data are expressed as the mean (SD)).

	Gender	
	Males	Females
**Age, y**	10.2 (2.69)	10.2 (3.35)
**Weight, kg**	62.2 (16.2)	53.7 (18.81)
**Weight z-score**	10.2 (2.69)	10.2 (3.35)
**Height, m**	1.5 (0.14)	1.4 (0.19)
**Height z-score**	2.7 (1.09)	2.6 (1.11)
**BMI kg/m^2^**	27.2 (3.18)	26.9 (4.22)
**BMI z-score**	2.75 (0.7)	2.73 (0.6)
**Waist, cm**	90.4 (10.67)	85.6 (10.92)
**Waist/Height**	3.1 (0.92)	3 (1.08)

**Table 2:** Correlation matrix between VitD serum values and adiposity indices.

	VitD	BMI	Waist	W/H	Weight
VitD	1				
BMI	−0.2430 **	1			
Waist	−0.2390 *	0.6889 **	1		
W/H	−0.0413	0.2962 **	0.3415 **	1	
Weight	−0.3058 **	0.7987 **	0.8310 **	−0.02	1

* *p*-value < 0.05; ** *p*-value < 0.01.

**Table 3:** Description of questionnaire scores by vitamin D status, considering serum levels of both 30 and 20 ng/mL as cut-offs for VitD insufficiency.

**VitD Cut-Off 30 ng/mL**	**Number of Patients**	**Questionnaire Score**		
		**Mean (SD)**	**Median**	**Min–Max**
Severe deficiency (<10)	19	20.7 (4.26)	19.3	14–27
Insufficiency (10–30)	97	20.4 (3.24)	17	15–30
Sufficiency (>30)	4	19.8 (3.3)	0	15–22
**VitD Cut-Off 20 ng/mL**	**Number of Patients**	**Questionnaire Score**		
		**Mean (SD)**	**Median**	**Min–Max**
Severe deficiency (<10)	19	20.7 (4.26)	21	14–27
Insufficiency (10–20)	50	20.2 (3.27)	19	15–28
Sufficiency (>20)	51	20.5 (3.21)	20	15–30

**Table 4:** Questionnaire score cut-off to discriminate between subjects with severe VitD deficiency and those with VitD sufficiency according to VitD levels.

Score Cut-Off	Sensitivity	95% CI	Specificity	95% CI	#Pos	#Neg
**Severe Deficiency 25(OH)D < 10 mg/dL**						
30	0.00%	(0–0.209)	99.01%	(0.938–0.999)	0	1
27	15.79%	(0.042–0.405)	96.04%	(0.896–0.987)	3	3
21 ^a^	63.16%	(0.386–0.828)	55.45%	(0.452–0.652)	9	41
14	100.00%	(0.791–1)	0.00%	(0–0.046)	7	56
Severe deficiency prevalence (score ≥ 21) = 47.5%; ROC curve AUC = 0.615; SE = 0.082						
**Deficiency 25(OH)D < 20 mg/dL**						
14	4.35%	(0.011–0.13)	100.00%	(0.913–1)		
15	7.25%	(0.027–0.168)	98.04%	(0.882–0.999)	5	1
**19 ^a^**	**49.28%**	**(0.371–0.615)**	**64.71%**	**(0.5–0.772)**	**29**	**17**
28	100.00%	(0.934–1)	1.96%	(0.001–0.118)	18	19
30	100.00%	(0.934–1)	0.00%	(0–0.087)	35	33
Deficiency prevalence (score ≥ 19) = 69.2%; ROC curve AUC = 0.583; SE = 0.053						
**Insufficiency 25(OH)D < 30 mg/dL**						
23 ^a^	24.14%	(0.169–0.331)	100.00%	(0.396–1)	111	3
16	95.69%	(0.897–0.984)	25.00%	(0.013–0.781)	5	1
14	100.00%	(0.96–1)	0.00%	(0–0.604)	111	3
Insufficiency prevalence (score ≥ 23) = 23.3%; ROC curve AUC = 0.694; SE = 0.147						
**Sufficiency 25(OH)D ≥ 30 mg/dL**						
14	0.00%	(0–0.604)	97.41%	(0.921–0.993)	0	3
15	25.00%	(0.013–0.781)	95.69%	(0.897–0.984)	1	2
22 ^a^	100.00%	(0.396–1)	24.14%	(0.169–0.331)	0	0
30	100.00%	(0.396–1)	0.00%	(0–0.04)	3	111
Sufficiency prevalence (score ≥ 22) = 29.2%; ROC curve AUC = 0.691; SE = 0.147						

#Pos = positive predicted value; #Neg = negative predicted value; ^a^ = best cut-off for score predicting VitD shortage = 20.

**Table 5:** ROC analysis results using the 4 reference variables.

	25(OH)D < 10 mg/dL	25(OH)D < 20 mg/dL	25(OH)D < 30 mg/dL	25(OH)D ≥ 30 mg/dL
**Optimal operating slope**	1	1	1	1
**Optimal cut-off**	21	19	23	22
**Optimal sensitivity**	0.632	0.493	0.241	1
**Optimal specificity**	0.554	0.647	1	0.241
**Clinical information statistic**	0.186	0.139	0.241	0.241
**Area under the ROC Curve**	0.615	0.583	0.694	0.691
**SE of Area (Hanley)**	0.082	0.053	0.147	0.147
**Sample size**	120	120	120	120

## Data Availability

Data are contained within the article.
